# Linking tuberous sclerosis complex, excessive mTOR signaling, and age-related neurodegeneration: a new association between *TSC1* mutation and frontotemporal dementia

**DOI:** 10.1007/s00401-017-1764-0

**Published:** 2017-08-21

**Authors:** Nicholas T. Olney, Carolina Alquezar, Eliana Marisa Ramos, Alissa L. Nana, Jamie C. Fong, Anna M. Karydas, Joanne B. Taylor, Melanie L. Stephens, Andrea R. Argouarch, Victoria A. Van Berlo, Deepika R. Dokuru, Elliott H. Sherr, Gregory A. Jicha, William P. Dillon, Rahul S. Desikan, Mary De May, William W. Seeley, Giovanni Coppola, Bruce L. Miller, Aimee W. Kao

**Affiliations:** 1 Department of Neurology, Memory and Aging Center, University of California, Box 1207, San Francisco, CA 94158 USA; 2 Department of Neurology and Department of Psychiatry, Semel Institute for Neuroscience and Human Behavior, University of California, Los Angeles, CA 90095 USA; 3 Department of Neurology and Pediatrics, University of California, San Francisco, CA 94158 USA; 4 Department of Neurology, Sanders-Brown Center on Aging, University of Kentucky, Lexington, KY 40536 USA; 5 Department of Radiology, University of California, San Francisco, CA 94158 USA

A 52-year-old right-handed man presented for the evaluation of progressive emotional flattening, social withdrawal, and loss of empathy. Prior to symptom onset, his wife of 21 years described him as “easy going, loving, kind, and generous.” As symptoms progressed, the subject began to alienate coworkers with unwelcome and persistent practical jokes. These disinhibited behaviors were accompanied by new, compulsive Internet usage. No language, visuospatial, memory, or motor symptoms were reported.

Past medical history included chronic headaches and multiple concussions but no seizures, psychiatric disorders, or drug use. He denied cutaneous, dental, or renal abnormalities. His childhood development was unremarkable. He completed 13 years of formal education and worked in the upper echelons of management at a professional firm.

The subject’s family history (Supplementary Fig. S1a) was notable for a father with multiple concussions and late-life seizures, alcoholism, impulsiveness, and poor decision-making. An older sibling with left arm hemihypertrophy (Parkes-Weber Syndrome) was deceased after post-operative stroke. A younger sibling developed epilepsy at age 19 and underwent frontal lobe resection surgery at age 43; this individual experienced progressive difficulty with concentration and multitasking, qualifying for a diagnosis of mild cognitive impairment (MCI) in the executive domain.

On exam, the proband was 72 inches tall and weighed 220 lb (BMI of 29.8). Skin exam revealed ungual fibromas and a shagreen patch. His neurological exam was normal except for an expansive mood, intrusive commentary, and jocularity verging on the inappropriate. Neuropsychological testing revealed impaired confrontational naming with mild deficits in semantic knowledge. Visual episodic memory, executive functioning, and information processing speed were also impaired. Other cognitive domains were within normal limits for age. Deficits localized to the right greater than left temporal and frontal lobes. Magnetic resonance imaging (MRI) revealed corresponding areas of atrophy and white matter lesions (Fig. 1a). Electroencephalogram (EEG) was negative for epileptiform activity.

**Fig. 1:**
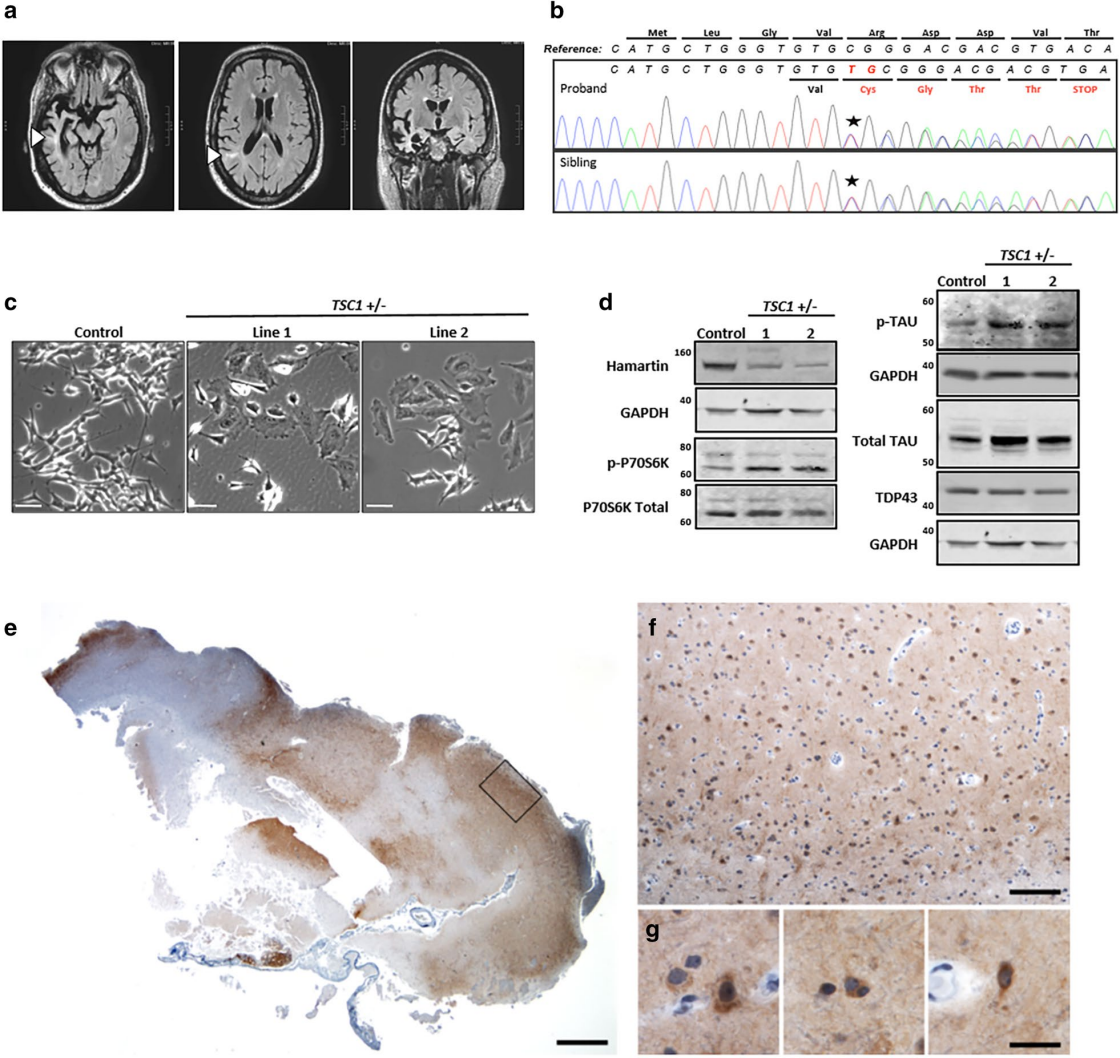
Proband clinical MRI, novel *TSC1* mutation, cell model of TSC1/hamartin haploinsufficiency, and neuropathology of a *TSC1* mutation carrier. **a** Magnetic resonance imaging (MRI) FLAIR sequences revealed severe atrophy of the right anterior temporal lobe, particularly the entorhinal/perirhinal cortices and inferior temporal gyrus, as well as the hippocampus and amygdala. There was also involvement of the left anterior/mesial temporal lobe and the bilateral frontal and right parietal lobes. T2/FLAIR hyperintensities are seen within the subcortical white matter, some of which extended to the superolateral aspects of the lateral ventricles (arrowheads). These lesions can be seen in tuberous sclerosis or focal cortical dysplasias as a transmantle sign. **b** Sanger sequencing confirmed a novel frameshift variant in the *TSC1* gene (NM_000368.4: c.62_63insTG: p. Arg22CysfsTer5) that segregated with the proband and affected sibling. **c** Representative micrographs of the enlarged cell body in the undifferentiated *TSC1* heterozygous mutant SH-SY5Y lines. *Scale bars* represent 50 microns. **d** Representative western blots showing decreased TSC1/hamartin and increased phopho-P70S6K^thr389^, total tau, and phospho-tau levels in retinoic acid differentiated *TSC1* +/− SH-SY5Y cells. TDP-43 levels are unchanged. **e**–**g** Fragment of frontal cortex resection from the proband’s sibling was received and stained for hyperphosphorylated tau, revealing patchy staining in neurons, glia, and surrounding neuropil. *Scale bars* represent 1000 (**e**), 250 (**f**), and 25 (**g**) microns

The proband met criteria for probable behavioral variant frontotemporal dementia (bvFTD), a sub-type of frontotemporal lobar degeneration (FTLD), based on early behavioral disinhibition, loss of empathy, perseverative behaviors, neuropsychological profile, and MRI findings [12]. Given his family history, he was screened for known genetic causes of FTLD with no observed pathogenic variants. Therefore, the proband and his affected sibling underwent whole-exome sequencing. Among variants identified, a novel frameshift mutation in one allele of the *TSC1* gene, predicted to result in early termination of the TSC1/hamartin protein, was shared by the proband and his younger sibling (Fig. 1b, Supplementary Fig. S1b, c; Table S1).

TSC1/hamartin is an upstream inhibitor of mTOR, which regulates cell growth and protein degradation [8]. To determine the significance of the *TSC1* loss of function variant, CRISPR/Cas9 genome editing [6] was used to generate a cellular model of TSC1/hamartin deficiency in SH-SY5Y cells, a human neuroblastoma cell line that can be differentiated into neuronal-like cells [7] (Supplementary Fig. S2a). We confirmed that these lines exhibited decreased TSC1/hamartin, increased cell size, and elevated P70S6K phosphorylation, consistent with mTOR over activity (Fig. 1c, Supplementary Fig. S2b, c). After retinoic acid (RA) differentiation, *TSC1* +/− cells maintained lower levels of TSC1/hamartin and increased mTOR activity. Interestingly, the RA-differentiated *TSC1* +/− lines accumulated phospho-tau and total tau. However, levels of TDP-43, another protein that aggregates in FTLD [2], were unchanged compared to control (Fig. 1d, Supplementary Fig. S2d–h).

Histological samples were obtained from the partial frontal lobe resection of the affected sibling, also a *TSC1* mutation carrier. Similar to SH-SY5Y model of *TSC1* heterozygosity, immunostaining of the resected tissue for phosphorylated tau revealed patchy neuropil staining as well as diffuse cytoplasmic staining of scattered neurons and glia, with variable intensity (Fig. 1e–g). No neurofibrillary tangles, Pick bodies, glial inclusions, or other features of an inherited or sporadic tauopathy were observed. Sections were also stained for TDP-43, phospho-TDP-43, amyloid beta (Aβ), α-synuclein, and ubiquitin. Surprisingly, they did not demonstrate elevated immunoreactivity for any other neurodegenerative disease proteins (data not shown).

In this case report, we describe the first case of bvFTD in an adult with sub-clinical tuberous sclerosis complex (TSC) due to a novel *TSC1* frameshift variant. Although TSC has been called an “infantile tauopathy” [13], *TSC1* mutations have not previously been implicated in age-associated neurodegenerative disease. This report, therefore, potentially adds *TSC1* to the list of genes that are implicated in both a juvenile-onset lysosomal storage disease and adult-onset neurodegeneration. Others include *GBA* and *ATP13A2* in Parkinson’s disease, *CTSD* in Alzheimer’s disease, and *PGRN* in FTLD [10, 15]. The TSC1/TSC2 complex normally inhibits mTOR activity, with loss of function mutations resulting in overactive mTOR and consequential increased protein synthesis and decreased protein degradation [3, 8, 9]. Strikingly, the decreased protein degradation seems selective for tau, as evidenced from both our cell-based and neuropathological data. This case now suggests that with age, accumulated tau can precipitate neuron loss and neurodegeneration. It also raises the intriguing possibility that tau metabolism is selectively regulated by the mTOR pathway. Our data suggest that *TSC1* loss of heterozygosity, which is necessary for TSC-related tumors [11], is not required for *TSC1*-related tau accumulation and neurodegeneration.

Our findings could be highly significant to the care of TSC patients. Many features of tuberous sclerosis associated neuropsychiatric disorders (TAND) [4, 5], including obsessive behavior, attention deficits, altered eating, impulsivity, memory loss, and language dysfunction, overlap with bvFTD criteria. Thus, these two clinical entities may represent overlapping disorders precipitated by progressive tau pathology. Additional sequencing of the *TSC1/2* genes in FTLD cohorts, use of new tau-based PET imaging, and review of neuropathological specimens from older TSC patients may further support the association between TSC, TAND, and FTLD.

This study extends the relationship between mTOR and tau metabolism [1, 16] to include a potential age-related component. How does aging alter the TSC1/mTOR axis to potentially contribute to the development of neurodegeneration? Why do tau and phospho-tau accumulate, while related proteins that undergo chaperone mediated autophagy, such as TDP-43, do not? Do *TSC2* mutations also link to age-related tauopathies? These questions and others are stimulated by our report. In the meantime, this study supports trials of rapamycin analogs which are already underway for neurodegeneration [14], and moves us towards a personalized medicine future in which sub-populations of tauopathy patients may be selectively responsive to this therapeutic approach.

## Electronic supplementary material

Below is the link to the electronic supplementary material.

Supplementary material 1 (pdf 1253 kb)
