# Supplementary material for: Linking tuberous sclerosis complex, excessive mTOR signaling, and age-related neurodegeneration: a new association between TSC1 mutation and frontotemporal dementia
Source: Acta Neuropathol. 2017 Aug 21;134(5):813–6. doi: 10.1007/s00401-017-1764-0 (PMC5645431; doi:10.1007/s00401-017-1764-0)
Supplement: Supplementary file 1 — Supplementary material 1 (pdf 1253 kb) [file 401_2017_1764_MOESM1_ESM.pdf]

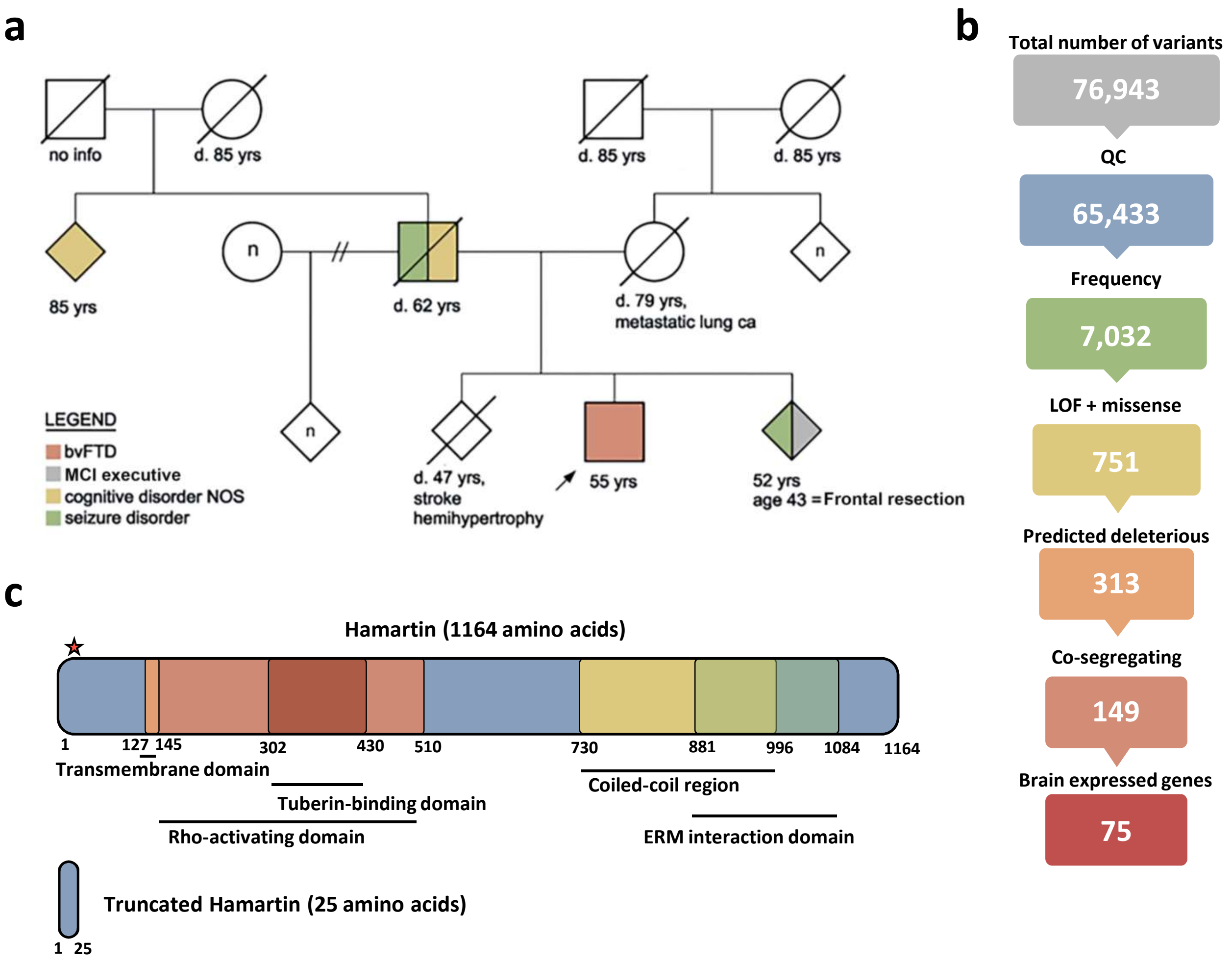

Supplementary figure 1.

Proband genetics. (**a**) Family pedigree showed multiple family members affected with cognitive and/or seizure disorder. If known, cause of death is noted. Arrow pointing the proband. n=indefinite number of family members. (**b**) A total of 76,943 variants were identified within the targeted exomes of the proband and living sibling. Variants of low quality were removed through quality control (CQ>20,RD>10, AF>20,>top 1% most exonically variable genes), followed by filtering of those with minor allele frequency (<1% in the 1000 Genomes Project, Exome Variant Server and >0.5% in Exome Aggregation Consortium databases). Analysis and filtering then focused on predicted deleterious missense, splice-site, start/stop codon change and indel variants. Of these, only 75 variants segregated with disease and were within genes expressed in the brain. The majority were predicted deleterious missense and in-frame indels, and 20 were predicted loss-of-function (LoF) variants, including *TSC1*. (**c**) The novel frameshift variant in the *TSC1* gene is predicted to result in a truncated 25 amino acid form of the TSC1/hamartin protein.

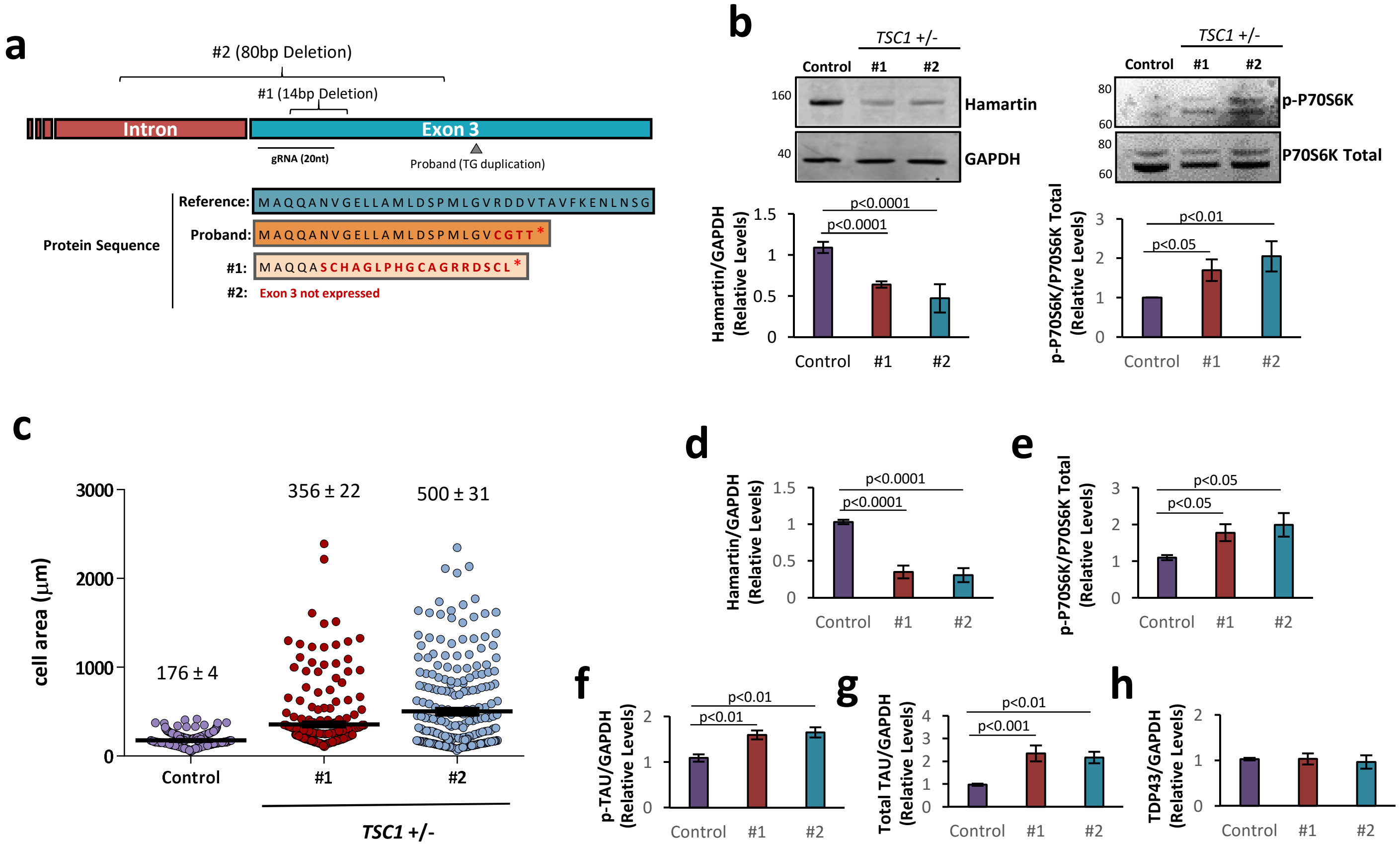

Supplementary figure 2.

Cell model of TSC1 haploinsufficiency exhibits elevated mTOR signaling and increased tau levels and phosphorylation. (**a**) A targeted single RNA guide (gRNA) against exon 3 of the *TSC1* gene (5'- GGCCCAACAAGCAAATGTCG-3') was introduced into the LentiCRISPRv2 (Addgene 52961) vector. Two *TSC1* heterozygous clones were generated. (**b**) Representative immunoblots show decreased TSC1/hamartin and increased phopho-P70S6K^{Thr398} in *TSC1* mutant clones. (**c**) Scatter plot representing the cell body area measurements of 300 independent cells for each cell line. (**d-h**) Quantification of the western blots showing decreased TSC1/hamartin and increased phopho-P70S6K^{Thr398}, total tau and phospho-tau in retinoic acid-differentiated *TSC1* +/- SH-SY5Y cells. TDP-43 levels are unchanged. Four biological replicates were carried out for each experiment and results were averaged for quantification. All the statistical data are presented as mean ± standard error of the mean (SEM). A value of p<0.05 was considered significant.

Supplementary Table 1. Genomic variants identified by Whole-Genome sequencing in the *TSC1* mutation carriers

Chromosome	Position	Reference Allele	Sample Allele	Gene	Transcript ID	Transcript Variant	Protein Variant	Translation Impact	SIFT Function Prediction	PolyPhen-2 Function Prediction	dbSNP ID	1000 Genomes Frequency	NHLBI ESP Frequency	ExAC Frequency	Brain Expressed
1	1684347	C	CCCT	NADK	NM_001198994	c.1771_1772insAGG	p.G591delinsEG	nonframeshift_insertion			150880809				yes
1	104076466	G	GA	RNPC3	NM_017619	c.347dupA	p.E116fs	frameshift_insertion			748882456				yes
1	117142868	C	T	IGSF3	NM_001542	c.G1784A	p.W595X	stop gain			61730489				yes
1	117156459	C	T	IGSF3	NM_001542	c.G760A	p.D254N	nonsynonymous	Damaging	Probably Damaging	61786651				yes
1	117156584	T	C	IGSF3	NM_001542	c.A635G	p.Q212R	nonsynonymous	Damaging	Possibly Damaging	143106517			0	yes
1	117156585	G	A	IGSF3	NM_001542	c.C634T	p.Q212X	stop gain			139013364			0	yes
1	207195437	G	A	C1orf116	NM_023938	c.C1672T	p.R558C	nonsynonymous	Damaging	Possibly Damaging	149974398	0.08	0.12	0.05	yes
1	207245631	G	A	PFKFB2	NM_006212	c.G1433A	p.R478H	nonsynonymous		Possibly Damaging	779972554			0	yes
2	18767638	A	G	NT5C1B	NM_001199087	c.T371C	p.L124P	nonsynonymous		Possibly Damaging	147855918	0.2	0.46	0.4	
2	27461368	T	C	CAD	NM_004341	c.T4930C	p.F1644L	nonsynonymous	Damaging	Probably Damaging	144692793	0.22	0.56	0.45	yes
2	29074141	A	G	TRMT61B	NM_017910	c.T1109C	p.L370S	nonsynonymous	Damaging	Probably Damaging	150890124	0.04	0.29	0.19	yes
2	60689202	A	G	BCL11A	NM_022893	c.T845C	p.L282P	nonsynonymous	Damaging	Probably Damaging	377282022		0.01		yes
2	96517893	T	C	ANKRD36C	NM_001310154	c.6298A>G	p.M2100V	nonsynonymous			77129683			0.01	
2	96517905	A	T	ANKRD36C	NM_001310154	c.6286T>A	p.C2096S	nonsynonymous			80041617			0	
2	96521334	T	C	ANKRD36C	NM_001310154	c.5773A>G	p.M1925V	nonsynonymous			201192250			0.08	
2	96521512	A	C	ANKRD36C	NM_001310154	c.5595T>G	p.C1865W	nonsynonymous			78536198				
2	98128313	G	A	ANKRD36B	NM_025190	c.C3008T	p.T1003M	nonsynonymous	Damaging	Probably Damaging					yes
2	133070449	T	C	ZNF806	NM_001304449	c.23T>C	p.V8A	nonsynonymous			2459647				
2	133070508	G	T	ZNF806	NM_001304449	c.82G>T	p.A28S	nonsynonymous			7340197				
2	133070556	T	C	ZNF806	NM_001304449	c.130T>C	p.F44L	nonsynonymous			7355766				
2	133074876	A	G	ZNF806	NM_001304449	c.337A>G	p.M113V	nonsynonymous			7355689				
2	133075027	G	A	ZNF806	NM_001304449	c.488G>A	p.G163E	nonsynonymous			7349198				
2	133075365	G	A	ZNF806	NM_001304449	c.826G>A	p.V276I	nonsynonymous			7349215				
2	133075386	G	A	ZNF806	NM_001304449	c.847G>A	p.G283R	nonsynonymous			7349216				
2	133075478	TC	T	ZNF806	NM_001304449	c.942delC	p.Y315fs*152	frameshift_deletion			111944984				
2	133075612	C	T	ZNF806	NM_001304449	c.1073C>T	p.P358L	nonsynonymous			11491243				
2	133075615	G	C	ZNF806	NM_001304449	c.1076G>C	p.C359S	nonsynonymous			76315261				
2	133075639	G	T	ZNF806	NM_001304449	c.1100G>T	p.G367V	nonsynonymous			7340191				
2	133075664	T	G	ZNF806	NM_001304449	c.1125T>G	p.D375E	nonsynonymous			7340499				
2	133075674	G	C	ZNF806	NM_001304449	c.1135G>C	p.G379R	nonsynonymous			7340192				

Supplementary Table 1. Genomic variants identified by Whole-Genome sequencing in the *TSC1* mutation carriers (Continuation)

Chromosome	Position	Reference Allele	Sample Allele	Gene	Transcript ID	Transcript Variant	Protein Variant	Translation Impact	SIFT Function Prediction	PolyPhen-2 Function Prediction	dbSNP ID	1000 Genomes Frequency	NHLBI ESP Frequency	ExAC Frequency	Brain Expressed
2	133075726	A	G	ZNF806	NM_001304449	c.1187A>G	p.E396G	nonsynonymous			2598810				
2	133076032	T	G	ZNF806	NM_001304449	c.1493T>G	p.F498C	nonsynonymous			2598809				
2	133076117	CA	C	ZNF806	NM_001304449	c.1580delA	p.N527fs*36	frameshift_deletion			111405036				
2	133076155	G	A	ZNF806	NM_001304449	c.1616G>A	p.C539Y	nonsynonymous			2677622	0.04			
2	133076206	C	T	ZNF806	NM_001304449	c.1667C>T	p.A556V	nonsynonymous			2598807				
2	133076221	G	A	ZNF806	NM_001304449	c.1682G>A	p.R561H	nonsynonymous			7340213				
2	133076229	A	G	ZNF806	NM_001304449	c.1690A>G	p.K564E	nonsynonymous			2598806				
2	179341900	C	G	FKBP7	NM_181342	c.G262C	p.G88R	nonsynonymous	Damaging	Probably Damaging	202240908	0.02	0.01	0.01	yes
3	75714805	TG	T	FRG2C	NM_001124759	c.463delG	p.G155fs	frameshift_deletion			144577984				
3	75714807	G	A	FRG2C	NM_001124759	c.G464A	p.G155E	nonsynonymous	Damaging	Probably Damaging	62247158				
3	75714950	C	A	FRG2C	NM_001124759	c.C607A	p.L203M	nonsynonymous	Damaging	Probably Damaging	73840338				
3	75715124	C	T	FRG2C	NM_001124759	c.C781T	p.P261S	nonsynonymous		Probably Damaging	72503535				
3	75715173	C	T	FRG2C	NM_001124759	c.C830T	p.A277V	nonsynonymous		Probably Damaging	199538970				
3	75715181	G	A	FRG2C	NM_001124759	c.G838A	p.G280R	nonsynonymous		Probably Damaging	201780087				
3	75779769	C	G	ZNF717	NM_001290210	c.G340C	p.V114L	nonsynonymous			72503549				
3	75781222	C	T	ZNF717	NM_001290210	c.G328A	p.E110K	nonsynonymous			62250084				
3	75786031	A	C	ZNF717	NM_001290209	c.T2593G	p.X865G	stop loss			200852928				
3	75786035	G	GA	ZNF717	NM_001290209	c.2588dupT	p.F863fs	frameshift_insertion			149076283				
3	75786252	G	T	ZNF717	NM_001290209	c.C2372A	p.P791H	nonsynonymous	Damaging	Probably Damaging	140641854			0.29	
3	75786278	A	C	ZNF717	NM_001290209	c.T2346G	p.H782Q	nonsynonymous	Damaging	Possibly Damaging	79811623				
3	75786288	A	G	ZNF717	NM_001290209	c.T2336C	p.L779P	nonsynonymous	Damaging	Probably Damaging	76707683				
3	75786314	CCTACATTCT	C	ZNF717	NM_001290209	c.2301_2309del	p.767_770del	nonframeshift_deletion			146447046				
3	75786417	T	C	ZNF717	NM_001290209	c.A2207G	p.Y736C	nonsynonymous	Damaging	Probably Damaging	77452946				
3	75786518	A	C	ZNF717	NM_001290209	c.T2106G	p.H702Q	nonsynonymous	Damaging	Probably Damaging	113078821				
3	75786773	T	A	ZNF717	NM_001290209	c.A1851T	p.R617S	nonsynonymous	Damaging	Possibly Damaging	76175438				
3	75786827	A	T	ZNF717	NM_001290209	c.T1797A	p.C599X	stop gain			79635065				
3	75787042	G	GAAAT	ZNF717	NM_001290209	c.1581_1582insATTT	p.L528fs	frameshift_insertion			369928577				
3	75787044	A	ACTT	ZNF717	NM_001290209	c.1579_1580insAAG	p.F527delinsX	stop gain			373780316				
3	75787321	C	T	ZNF717	NM_001290209	c.G1303A	p.G435R	nonsynonymous	Damaging	Probably Damaging	202223162			0	
3	75787516	C	G	ZNF717	NM_001290209	c.G1108C	p.E370Q	nonsynonymous	Damaging	Possibly Damaging	79870536				

Supplementary Table 1. Genomic variants identified by Whole-Genome sequencing in the *TSC1* mutation carriers (Continuation)

Chromosome	Position	Reference Allele	Sample Allele	Gene	Transcript ID	Transcript Variant	Protein Variant	Translation Impact	SIFT Function Prediction	PolyPhen-2 Function Prediction	dbSNP ID	1000 Genomes Frequency	NHLBI ESP Frequency	ExAC Frequency	Brain Expressed
3	75787519	C	A	ZNF717	NM_001290209	c.G1105T	p.G369W	nonsynonymous	Damaging	Probably Damaging	200805142				
3	75787645	GAA	G	ZNF717	NM_001290209	c.977_978del	p.F326fs	frameshift_deletion			141065192				
3	75787732	A	C	ZNF717	NM_001290209	c.T892G	p.F298V	nonsynonymous	Damaging	Possibly Damaging	74776730				
3	75787996	C	T	ZNF717	NM_001290209	c.G628A	p.G210R	nonsynonymous	Damaging	Possibly Damaging	78640256				
3	75788028	G	C	ZNF717	NM_001290209	c.C596G	p.S199X	stop gain			77971486				
3	75788150	A	AG	ZNF717	NM_001290209	c.473dupC	p.T158fs	frameshift_insertion			143871834				
3	147123200	G	T	ZIC4	NM_001168379	c.C59A	p.S20X	stop gain							yes
3	183493743	CGGA	C	YEATS2	NM_018023	c.2410_2412del	p.804_804del	nonframeshift_deletion			146705467				yes
3	186395177	CCATGGA	C	HRG	NM_000412	c.1084_1089del	p.362_363del	nonframeshift_deletion							
4	3138013	G	A	HTT	NM_002111	c.G2758A	p.D920N	nonsynonymous			80100842	0.02	0.06	0.03	yes
4	25665907	G	A	SLC34A2	NM_006424	c.G334A	p.V112M	nonsynonymous		Probably Damaging	78448446			0	yes
4	126238090	G	T	FAT4	NM_001291285	c.G524T	p.R175L	nonsynonymous		Probably Damaging	143534324	0.1	0.36	0.3	yes
5	41909884	C	A	C5orf51	NM_175921	c.C244A	p.L82I	nonsynonymous	Damaging	Probably Damaging	142045369	0.34	0.41	0.4	yes
5	68692375	T	TA	RAD17	NM_133339	c.1605+2insA		splice site			377737971				yes
5	75960968	G	C	IQGAP2	NM_006633	c.G2647C	p.E883Q	nonsynonymous	Damaging	Probably Damaging	34968964	0.12	0.41	0.34	yes
5	118484637	A	T	DMXL1	NM_001290321	c.A3115T	p.S1039C	nonsynonymous	Damaging	Possibly Damaging	149431940	0.16	0.15	0.2	yes
5	122152641	G	A	SNX2	NM_003100	c.G830A	p.S277N	nonsynonymous	Damaging	Possibly Damaging					yes
5	140772573	C	T	PCDHGA8	NM_014004	c.C193T	p.R65C	nonsynonymous	Damaging	Probably Damaging	182052960	0.36	0.21	0.23	
6	7910870	T	TCCG	TXNDC5	NM_030810	c.139_140insCGG	p.D47delinsAD	nonframeshift_insertion			759591654				yes
6	54185433	A	C	TINAG	NM_014464	c.A412C	p.N138H	nonsynonymous	Damaging	Probably Damaging	773597964			0	yes
6	73951871	G	A	KHDC1	NM_001251874	c.C421T	p.R141C	nonsynonymous	Damaging		773192554			0	
8	49643960	C	T	EFCAB1	NM_024593	c.G161A	p.R54Q	nonsynonymous	Damaging	Probably Damaging	74697155	0.38	0.55	0.44	yes
8	95201468	G	A	CDH17	NM_001144663	c.C97T	p.P33S	nonsynonymous	Damaging	Possibly Damaging	145693879	0.02	0.15	0.11	
8	98991158	T	C	MATN2	NM_002380	c.T1003C	p.C335R	nonsynonymous	Damaging	Probably Damaging	184482356	0.04	0.08	0.05	yes
9	32633036	C	T	TAF1L	NM_153809	c.G2542A	p.D848N	nonsynonymous	Damaging	Probably Damaging	141368669		0.08	0.08	
9	35847081	T	TG	TMEM8B	NM_016446	c.909dupG	p.L303fs	frameshift_insertion							yes
9	69423550	G	T	ANKRD20A4	NM_001098805	c.G1846T	p.A616S	nonsynonymous	Damaging	Possibly Damaging	200486187				
9	69423844	C	G	ANKRD20A4	NM_001098805	c.C2140G	p.Q714E	nonsynonymous	Damaging	Possibly Damaging	201501857				
9	69423863	T	A	ANKRD20A4	NM_001098805	c.T2159A	p.V720E	nonsynonymous	Damaging	Possibly Damaging	202042730				
9	117848668	G	C	TNC	NM_002160	c.C1342G	p.R448G	nonsynonymous	Damaging	Possibly Damaging	145086096	0.2	0.49	0.44	yes

Supplementary Table 1. Genomic variants identified by Whole-Genome sequencing in the *TSC1* mutation carriers (Continuation)

Chromosome	Position	Reference Allele	Sample Allele	Gene	Transcript ID	Transcript Variant	Protein Variant	Translation Impact	SIFT Function Prediction	PolyPhen-2 Function Prediction	dbSNP ID	1000 Genomes Frequency	NHLBI ESP Frequency	ExAC Frequency	Brain Expressed
9	135804196	G	GCA	TSC1	NM_000368	c.62_63insTG	p.R22fs	frameshift_insertion							yes
10	16737156	T	TG	RSU1	NM_012425	c.599-2insC		splice site			373104238				yes
10	126683123	A	C	CTBP2	NM_022802	c.T2315G	p.L772W	nonsynonymous	Damaging	Probably Damaging	79936509				yes
10	126727602	T	A	CTBP2	NM_001083914	c.A22T	p.K8X	stop gain			76555439			0	yes
11	6662745	C	CCAG	DCHS1	NM_003737	c.99_100insCTG	p.G34delinsLG	nonframeshift_insertion			376287018				yes
11	73007871	G	A	P2RY6	NM_001277208	c.G611A	p.R204H	nonsynonymous	Damaging	Probably Damaging	146746862	0.02	0.09	0.11	
12	7045891	A	ACAGCAG	ATN1	NM_001007026	c.1461_1462insCAGCAG	p.Q487delinsQQQ	nonframeshift_insertion							yes
12	55820958	CA	C	OR6C76	NM_001005183	c.922delA	p.K308fs	frameshift_deletion			57387180				
12	56500502	T	TA	PA2G4	NM_006191	c.217+2insA		splice site			34728522				yes
12	70747693	TA	T	CNOT2	NM_001199303	c.1622delA	p.X541X	nonframeshift_deletion			758089473				yes
12	123814987	A	C	SBNO1	NM_001167856	c.T1113G	p.I371M	nonsynonymous	Damaging	Possibly Damaging	140771058	0.02	0.02	0.02	yes
12	132401575	C	T	ULK1	NM_003565	c.C2150T	p.T717M	nonsynonymous	Damaging	Possibly Damaging	200524201	0.04	0.04	0.03	yes
13	21729952	T	TAA	SKA3	NM_145061	c.1120-2insTT		splice site							yes
13	39608335	T	TA	PROSER1	NM_025138	c.46-2insT		splice site			768981793				
13	100622667	TGGC	T	ZIC5	NM_033132	c.1260_1262del	p.420_421del	nonframeshift_deletion			746241424				yes
14	39784004	GTA	G	CTAGE5	NM_001247989	c.1371+2_1371+3delTA		splice site			75318507				yes
14	61482661	G	A	SLC38A6	NM_001172702	c.G350A	p.G117E	nonsynonymous	Damaging	Probably Damaging	749369988			0	yes
14	64908800	C	T	MTHFD1	NM_005956	c.C1913T	p.P638L	nonsynonymous	Damaging	Probably Damaging	149492308		0.02	0.01	yes
14	104206622	T	C	PPP1R13B	NM_015316	c.A2131G	p.I711V	nonsynonymous	Damaging	Probably Damaging					yes
15	85790360	C	T	GOLGA6L9	NM_001310153	c.1552C>T	p.R518W	nonsynonymous							
16	4519368	G	C	NMRAL1	NM_020677	c.C139G	p.L47V	nonsynonymous	Damaging	Possibly Damaging	779567114			0	yes
16	46958395	A	C	GPT2	NM_133443	c.A1307C	p.Q436P	nonsynonymous	Damaging	Probably Damaging					yes
16	47545670	A	G	PHKB	NM_000293	c.A500G	p.Y167C	nonsynonymous	Damaging	Probably Damaging	151155518	0.1	0.37	0.48	yes
16	58577315	GA	GAA	CNOT1	NM_206999	c.4628dupT	p.F1543fs	frameshift_insertion							yes
16	70500809	G	A	FUK	NM_145059	c.G436A	p.V146M	nonsynonymous	Damaging	Probably Damaging	17881323	0.08	0.3	0.21	yes
16	70884524	C	G	HYDIN	NM_001270974	c.G12478C	p.E4160Q	nonsynonymous	Damaging		1798314				yes
16	70894087	T	C	HYDIN	NM_001270974	c.A12013G	p.T4005A	nonsynonymous	Damaging		1539302				yes
16	70896015	GA	G	HYDIN	NM_001270974	c.11712delT	p.I3904fs	frameshift_deletion			77276171				yes
16	70954703	GGCGCTCCTTCTCCGT	G	HYDIN	NM_001270974	c.7561_7575del	p.2521_2525del	nonframeshift_deletion			67115747				yes
16	70989335	G	A	HYDIN	NM_001270974	c.C6259T	p.R2087C	nonsynonymous	Damaging		1774541	0.04			yes

Supplementary Table 1. Genomic variants identified by Whole-Genome sequencing in the *TSC1* mutation carriers (Continuation)

Chromosome	Position	Reference Allele	Sample Allele	Gene	Transcript ID	Transcript Variant	Protein Variant	Translation Impact	SIFT Function Prediction	PolyPhen-2 Function Prediction	dbSNP ID	1000 Genomes Frequency	NHLBI ESP Frequency	ExAC Frequency	Brain Expressed
16	71007809	C	T	HYDIN	NM_001270974	c.G5152A	p.V1718M	nonsynonymous	Damaging		783762				yes
16	71061495	A	G	HYDIN	NM_017558	c.T3052C	p.X1018Q	stop loss			1022220				yes
16	71098649	T	C	HYDIN	NM_001270974	c.A2170G	p.N724D	nonsynonymous	Damaging	Probably Damaging	3817211				yes
16	71122408	C	T	HYDIN	NM_001270974	c.G1466A	p.G489D	nonsynonymous	Damaging	Probably Damaging	62040318				yes
17	8701167	CA	C	MFSD6L	NM_152599	c.1271delT	p.L424fs	frameshift_deletion			772162369		0.15	0.2	
17	8701170	TGTA	T	MFSD6L	NM_152599	c.1266_1268del	p.422_423del	nonframeshift_deletion			773237450		0.14	0.2	
17	8701176	TTTG	T	MFSD6L	NM_152599	c.1260_1262del	p.420_421del	nonframeshift_deletion			766497350		0.14	0.2	
17	8722448	C	T	PIK3R6	NM_001010855	c.1948G>A	p.V650M	nonsynonymous		Probably Damaging	189806313	0.04	0.13	0.18	
17	17039561	CCAG	C	MPRIP	NM_015134	c.534_536del	p.178_179del	nonframeshift_deletion			3833098				yes
17	27010724	C	T	SUPT6H	NM_003170	c.C2119T	p.R707W	nonsynonymous	Damaging	Possibly Damaging	202102350	0.02		0	yes
17	45234360	A	C	CDC27	NM_001114091	c.T761G	p.L254X	stop gain			62077264				yes
18	48510894	C	T	ELAC1	NM_018696	c.C586T	p.R196C	nonsynonymous	Damaging	Probably Damaging	536968897			0	yes
18	72223591	G	GTGC	CNDP1	NM_032649	c.43_44insTGC	p.V15delinsVL	nonframeshift_insertion			10663835				yes
19	12243995	C	G	ZNF20	NM_021143	c.G1006C	p.E336Q	nonsynonymous	Damaging	Possibly Damaging	61745731	0.32	0.56	0.5	yes
19	19135760	G	A	SUGP2	NM_001017392	c.C1397T	p.A466V	nonsynonymous	Damaging	Probably Damaging					yes
19	41510282	A	G	CYP2B6	NM_000767	c.A415G	p.K139E	nonsynonymous	Damaging	Possibly Damaging	12721655	0.08	0.27	0.23	
19	42132274	G	A	CEACAM4	NM_001817	c.C125T	p.P42L	nonsynonymous	Damaging	Probably Damaging	148045742	0.12	0.12	0.18	yes
19	46299138	T	TCTCCTCGC CCTCCTCCTC	RSPH6A	NM_030785	c.2142_2143insGAG GAGGAGGGCGAGG AG	p.T715delinsEE EGEET	nonframeshift_insertion			773028484				yes
19	53668751	G	A	ZNF665	NM_024733	c.C992T	p.S331L	nonsynonymous	Damaging	Probably Damaging	200045588	0.04	0.1	0.05	
21	14987811	C	T	POTED	NM_174981	c.C730T	p.H244Y	nonsynonymous	Damaging	Probably Damaging	56121372				
21	14987871	G	T	POTED	NM_174981	c.G790T	p.D264Y	nonsynonymous	Damaging	Possibly Damaging	55653693				
21	30378845	TAACACTG	T	RWDD2B	NM_016940	c.846_852del	p.F282fs	frameshift_deletion			770677452		0.34	0.03	yes
21	31971187	C	T	KRTAP6-2	NM_181604	c.G7A	p.G3S	nonsynonymous		Possibly Damaging	150811972		0.02	0	yes
21	32253678	A	T	KRTAP11-1	NM_175858	c.T166A	p.C56S	nonsynonymous		Probably Damaging	149011340		0.01	0	
21	44324388	T	A	NDUFV3	NM_021075	c.1264+2T>A		splice site			145166807		0.04	0.09	yes
22	25010828	G	A	GGT1	NM_001288833	c.G250A	p.G84S	nonsynonymous	Damaging	Probably Damaging	77018131	0.02		0	yes
22	25011031	C	T	GGT1	NM_001288833	c.C319T	p.R107C	nonsynonymous	Damaging	Probably Damaging	142987478			0	yes
X	2833638	C	T	ARSD	NM_001669	c.G959A	p.G320D	nonsynonymous	Damaging	Probably Damaging	370769167			0.01	yes
X	55172537	G	A	FAM104B	NM_001166700	c.C331T	p.R111X	stop gain			1047054			0.05	yes

Materials and Methods:

Subjects

The protocol for this study was approved by the Institutional Review Board of the University of California, San Francisco. After informed consent was obtained, subjects underwent neurological evaluation, neuropsychological testing, informant interview, blood draw and neuroimaging.

Sequencing

The proband was screened by Sanger sequencing for mutations in the following genes: *APP, PSEN1, PSEN2, MAPT, PGRN, FUS, and TARDBP*. The hexanucleotide repeat length of *C9orf72* was screened as previously described [1].Whole exome regions of the proband and sibling were captured using the SeqCap EZ Human Exome Kit v3 and sequenced on an Illumina HiSeq2500 sequencer with an average of 115x depth of coverage. Sequence reads were mapped to the GRCh37/hg19 reference genome and variants joint-called using the GATK Haplotype Caller according to GATK Best Practices recommendations [2]. Ingenuity Variant Analysis was used for variant annotation and filtering. A series of filtering steps were applied to prioritize variants, as previously described [3]. PCR amplification from genomic DNA, followed by Sanger sequencing was performed to validate candidate variants.

Cell based assays

Generation of the TSC1 +/- cell lines

The SH-SY5Y neuroblastoma cell line (ATCC) was subjected to lentiviral CRISPR/Cas9 genome editing of the *TSC1* locus [4–6]. Targeted single RNA guide (gRNA) sequence against the exon 3 of the *TSC1* gene (5'-GGCCCAACAAGCAAATGTCG-3') was introduced into the LentiCRISPRv2 (addgene 52961) vector following the lentiviral CRISPR plasmid generation protocol [6].

Lentivirus particles were produced by co-transfection of the LentiCRISPRv2 plasmid containing the gRNA sequence with the packaging plasmids psPAX2 (AddGene 12260) and pCMV-VSV-G (AddGene 18454) into HEK296FT.

SH-SY5Y cells were infected with virus particles and, after the antibiotic selection, individual clones with enlarged cell size were isolated, expanded, checked by PCR and sequenced.

Cell area measurement

Micrographs from control and *TSC1* mutant cells (passage 3) were taken using a Carl Zeiss Axio Ver.A1 inverted microscope with 20x magnification. Measurement of cell area of 300 independent cells was performed using Image J. Statistical significance was estimated by one-way analysis of variance (ANOVA) followed by the Bonferroni's test for multiple comparisons. A value of $p<0.05$ was considered significant.

Cell differentiation

SH-SY5Y cell lines were differentiated into neurons by treatment with 10µM of retinoic acid (RA) for 6 days in EMEN/F12 media supplemented with 10% of FBS and 1% Penicillin-Streptomycin, followed by another 4 days of treatment with 50ng/mL of BDNF in EMEN/F12 media supplemented only with 1% Penicillin-Streptomycin [7].

Cell lysates and Western blotting

Cell lysates of non-differentiated and differentiated SH-SY5Y cells were collected using 1x RIPA buffer (Thermo Scientifics) supplemented with a cocktail of proteases and phosphatases inhibitors (Roche). Total protein concentration was quantified using Pierce™ BCA Protein Assay kit (Thermo Scientifics). Proteins were separated using Novex NuPage SDS-Page gel system (Thermo Scientifics followed by western blot using antibodies against TSC1/hamartin (1:500, Abcam #173410, Cambridge, MA, USA), P70S6K (1:1000, Cell Signaling #2708, Danvers, MA, USA), phospho-P70S6K^{thr389} (1:1000, Cell Signaling #9234, Danvers, MA, USA), phospho-Ser202-tau (AT8, 1:250, Thermo Scientifics #MN1020, Walthman, MA, USA), total tau (HT7, 1:250, Thermo Scientifics #PI206516, Walthman, MA, USA) and TDP-43 (1:1000, Proteintech #10782-2-AP, Rosemont, IL, USA). Imaging and quantification of the band intensity was performed on a LI-COR Odyssey Infrared System. At least three biological replicates were performed for each experiments and results were averaged for quantification. Statistical analyses were performed with Graph

Pad Prism 6 (La Jolla, CA, USA). All the statistical data are presented as mean ± standard error of the mean (SEM). Statistical significance was estimated by one-way analysis of variance (ANOVA) followed by the Bonferroni's test for multiple comparisons. A value of $p<0.05$ was considered significant.

Neuropathological studies

For neuropathological studies, archival formalin-fixed, paraffin embedded tissue sections from the living sibling's temporal lobe resection were made available. Sections were stained using antibodies against tau phosphorylated at serine 202 (CP-13 1:1000, gift from Peter Davies, Feinstein Institute, Manhasset, NY, USA), alpha-synuclein (1:1000, Millipore, Billerica, MA, USA), TDP-43 (1:4000, Proteintech Group, Chicago, IL, USA), amyloid beta (4G8, 1:250, Millipore, Billerica, MA, USA), and ubiquitin.

Bibliography

1. DeJesus-Hernandez M, Mackenzie IR, Boeve BF, et al (2011) Expanded GGGGCC Hexanucleotide Repeat in Noncoding Region of C9ORF72 Causes Chromosome 9p-Linked FTD and ALS. Neuron 72:245–256. doi: 10.1016/j.neuron.2011.09.011

2. McKenna A, Hanna M, Banks E, et al (2010) The Genome Analysis Toolkit: A MapReduce framework for analyzing next-generation DNA sequencing data. Genome Res 20:1297–1303. doi: 10.1101/gr.107524.110

3. Legati A, Giovannini D, Nicolas G, et al (2015) Mutations in XPR1 cause primary familial brain calcification associated with altered phosphate export. Nat Genet 47:579–581. doi: 10.1038/ng.3289

4. Doudna JA, Charpentier E (2014) The new frontier of genome engineering with CRISPR-Cas9. Science (80-) 346:1258096–1258096. doi: 10.1126/science.1258096

5. Sanjana NE, Shalem O, Zhang F (2014) Improved vectors and genome-wide libraries for CRISPR screening. Nat Methods 11:783–4. doi: 10.1038/nmeth.3047

6. Shalem O, Sanjana NE, Hartenian E, et al (2014) Genome-scale CRISPR-Cas9 knockout screening in human cells. Science 343:84–7. doi: 10.1126/science.1247005

7. Encinas M, Iglesias M, Liu Y, et al (2000) Sequential treatment of SH-SY5Y cells with retinoic acid and brain-derived neurotrophic factor gives rise to fully differentiated, neurotrophic factor-dependent, human neuron-like cells. J Neurochem 75:991–1003.
